# Medaka embryos as a model for metabolism of anabolic steroids

**DOI:** 10.1007/s00204-022-03284-4

**Published:** 2022-03-30

**Authors:** Lingyu Liu, Leonie Hobohm, Felix Bredendiek, Alexander Froschauer, Oliver Zierau, Maria Kristina Parr, Annekathrin M. Keiler

**Affiliations:** 1 Institute of Pharmacy, Pharmaceutical and Medicinal Chemistry, Freie Universität Berlin, Königin-Luise-Straße 2+4, 14195 Berlin, Germany; 2 Environmental Monitoring & Endocrinology, Faculty of Biology, Technische Universität Dresden, Zellescher Weg 20b, 01217 Dresden, Germany; 3 Core Facility BiosupraMol, Department of Biology, Chemistry, Pharmacy, Freie Universität Berlin, Berlin, Germany; 4 Institute of Doping Analysis & Sports Biochemistry, Dresdner Str. 12, 01731 Kreischa, Germany

**Keywords:** Doping analysis, Biotransformation, Metandienone, Mass spectrometry, *Oryzias latipes*, Fish embryo toxicity

## Abstract

In anti-doping science, the knowledge of drug metabolism is a prerequisite to identify analytical targets for the detection of misused prohibited substances. As the most obvious way to study xenobiotic metabolism, the administration to human volunteers, faces ethical concerns, there is a need for model systems. In the present study, we investigated whether *Oryzias latipes* (medaka) embryos might be an alternative, non-animal test model to study human-like metabolism. In the present study, we exposed medaka embryos at the morula stage to the anabolic steroid metandienone (10 µM or 50 µM) for a period of 2 or 8 days. According to the fish embryo toxicity test (OECD test), we assessed the developmental status of the embryos. We further investigated metandienone metabolites by high-performance liquid chromatography- and gas chromatography-mass spectrometry. Medaka embryos produced three mono-hydroxylated and one reduced metabolite known from human biotransformation. Developmental malformations were observed for the exposition to 50 µM metandienone, while a significant elevation of the heart beat was also present in those individuals exposed to the lower dose for 8 days. The present study demonstrates that the medaka embryo represents a promising model to study human-like metabolism. Moreover, the judgement of developmental parameters of the fish embryos enables for the simultaneous assessment of toxicity.

**Supplementary Information:**

The online version contains supplementary material available at 10.1007/s00204-022-03284-4.

## Introduction

The use and misuse of anabolic–androgenic steroids (AAS) has a variety of implications for society and the individual. Apart from the approved medical use, the anabolic (mis)use by athletes and non-athletes harbours the risk of adverse effects to the individual. Potential adverse effects of AAS use are diverse according to the dosage, steroid structure and simultaneous drug usage, amongst them liver damage, side effects on the cardiovascular system and the reproductive system (Büttner and Thieme 2010; Albano et al. 2021). In terms of sports doping testing according to the World Anti-Doping Agency’s Prohibited List, the most frequently detected substances are classified as AAS (World Anti-Doping Agency (WADA) 2020, 2021).

The AAS metandienone was developed in the mid-1950s and marketed as Dianabol^®^. Although its clinical approval was withdrawn in the early 1980s, metandienone is still marketed and used for performance enhancement. The metabolism of the orally bioavailable metandienone has been investigated thoroughly for decades, and there is still interest in finding new metabolites which might contribute to an elongation of the detection window (MacDonald et al. 1971; Schänzer et al. 1991, 2006; Pozo et al. 2009a; Loke et al. 2021; Parr et al. 2012). The most obvious way to study human xenobiotic metabolism is the administration to volunteers and the collection of their urine. Alternatively, laboratory animals like rodents, dogs or pigs are used as models for *in vivo* biotransformation studies (Zhang et al. 2012). Fishes are often described as “lower” vertebrates, ignoring their evolutionary success and their typical vertebrate bauplan. A fish model investigated for biotransformation studies of different compound classes with doping-relevance is the zebrafish (*Danio rerio*) (de Souza Anselmo et al. 2017; Sardela et al. 2018, 2020). In the so-called zebrafish water tank (ZWT) approach, test compounds are applied to the adult fish by solubilisation in the tank water. Those studies observed that the zebrafish is capable to generate and excrete human-like metabolites. As animal testing is strictly regulated in the European Union, each animal experiment including the treatment of adult fish requires approval by the responsible governing animal welfare authority. With regard to the 3R principle of animal welfare, the usage of fish embryos as alternative biotransformation model system is seen as a possibility to replace and reduce animal testing (Russel and Burch 1959). Fish embryos are considered as non-animal test system as they are excluded from animal testing regulations in toxicity studies (European Food Safety Authority (EFSA) 2005; The European Parliament and the Council of the European Union 2010). We have chosen the medaka (*Oryzias latipes*) for this pilot study, because of its ease in handling, the availability of embryos throughout the year, the excellent genomic and genetic resources and established protocols (Takeda and Shimada 2010). Medaka embryos produce 17ß-estradiol from administered testosterone indicating p450 aromatase activity early in development (Iwamatsu et al. 2006) and are also an already well-established species in environmental monitoring (Braunbeck et al. 2005; Horie et al. 2018).

The intention of this study was to investigate whether medaka embryos are an adequate alternative model for human biotransformation of doping-relevant compounds. For this purpose, we treated medaka embryos of different developmental stages with metandienone as model substance. Beside physiological parameters according to the OECD guidelines of the fish embryo acute toxicity test, metandienone metabolism was investigated applying gas chromatography- and liquid chromatography-mass spectrometric analyses.

## Materials and methods

### Chemicals and media

LGC Standards (Wesel, Germany) provided metandienone. β-Glucuronidase was purchased from Roche Diagnostics (Mannheim, Germany), and Testosterone-[d3] glucuronide from TRC (North York, USA). 17β-Hydroxy-17α-methyl-5β-androst-1-en-3-one and 6β-hydroxy metandienone were synthesized inhouse applying the procedures described by Schaenzer et al. (Schänzer et al. 1991, 1995).Sodium hydrogen phosphate, magnesium sulfate heptahydrate and DMSO (≥ 99.5%, BioScience Grade) were obtained from Carl Roth (Karlsruhe, Germany), sodium hydrogen carbonate and potassium carbonate from VWR (Darmstadt, Germany). N-Methyl-N-trimethylsilyl-trifluoroacetamide (MSTFA) was delivered from Chemische Fabrik Karl Bucher GmbH (Waldstetten, Germany), ethanethiol, ammonium iodide, formic acid (FoOH) and sodium chloride from Sigma Aldrich (Taufkirchen, Germany). Potassium dihydrogen phosphate was purchased from Merck (Darmstadt, Germany), while t-butyl methyl ether (TBME) was bought from Carl Roth (Darmstadt, Germany). Methanol and acetonitrile (ACN) were provided by Fisher Scientific (Loughborough, United Kingdom). Fluka (Buchs, Switzerland) provided potassium chloride and calcium chloride dihydrate. Embryo rearing medium (ERM) with final concentration of 1 g/l NaCl, 30 mg/l KCl, 40 mg/l CaCl_2_ × 2 H_2_O and 160 mg/l MgSO_4_ × 7 H_2_O were prepared from a 100 × stock solution with deionized water. Stock solutions of metandienone (50 mM and 10 mM) were prepared with DMSO.

### Fish strains

To test for sex-specific effects, we observed embryos of FLFII, in which the genotypic sex can be identified by the presence of leucophores in male embryos after 3 days of development (Wakamatsu et al., 2003). No sex-dependent differences in metabolite pattern were observed at 8 days of development in a pilot experiment, so we used embryos of mixed sexes (strain d-rR.YHNI, (Scholz et al. 2003)) for further experiments.

Animal husbandry, breeding and experiments involving fish embryos were conducted in the laboratories of the Environmental Monitoring & Endocrinology Group (Technische Universität Dresden, Germany). Administrative regulations and authorities (Landesdirektion Sachsen, File number 25-5131/346/6) approved the animal facility and procedures. The experiments described in this manuscript are no animal experimentation according to national law as well as the EU directive 2010/63/EU (The European Parliament and the Council of the European Union 2010).

### Exposition of fish embryos

Medaka embryos develop within 10 days from the fertilized egg to the 1st larval stage (hatching stage) (Iwamatsu 2004). We collected the eggs in the morning in embryo rearing medium and judged the egg quality by the observation of early life stages like the correct formation of blastomeres (Kinoshita et al. 2012). At stage 8 (morula), the embryos were sorted in the respective experimental groups. For exposition, up to 21 eggs were pooled in 5.5 cm glass petri-dishes and incubated at 26 °C on a shaker (5° tilt angle at 2–5 rpm). The petri-dishes were filled with 10 mL ERM and observed daily. Dead embryos were removed and the medium was changed every second day during the exposition.

In total, three independent experiments were conducted for MD exposition. In a pilot experiment, medaka embryos were exposed from morula stage onwards for 10 days with either 10 µM or 50 µM MD or with 0.1% DMSO (solvent control). Medium samples were collected every second day and analysed for metabolites (data not shown). In these experiments, a peak of metabolic capacity was identified after day 8 (8 dpf), that did not change until hatching. For the repetition of the metabolite assessments, we incubated medaka embryos with 10 µM MD for 48 h from 6 to 8 dpf. We did not repeat the experimental concentration of 50 µM MD due to the severe effects on embryonic development observed in the pilot screen.

### FET-assay

During exposition, parameters of the fish embryo toxicity (FET) assay were judged on Leica stereo microscopes (MZ8 for screening or F205FA with camera and Rottermann contrast for imaging). The FET parameters include normal development after blastula stage (stage10, after beginning of the experiment), formation of body axis, onset of blood circulation, hatching and other visible effects. On day 8, the developmental status was assessed for tail morphology and blood stream including the heart beat rate. Parameters are in accordance with OECD Test Guidelines 212/236 (OECD 1998, 2013). Specifically, we observed survival and defined developmental characteristics like angiogenesis, heart formation and general morphological parameters like head and tail morphology.

#### Statistical evaluation of heart beat

We used the Shapiro–Wilk test to judge whether the data describing heart beat frequency followed a normal distribution. When data were normally distributed (*p* value > 0.05), we applied an unpaired two-tailed Student’s *t *test to evaluate significant differences between MD treatment and respective control group. Statistical analysis and boxplots were made with R.

### Metabolite detection

To 1 mL of supernatant, 1 mL of phosphate buffer (pH 6.5), 50 μL of β-glucuronidase from *E. coli* and 100 μL of the internal standard (ISTD, Testosterone-[d3] glucuronide, 10 µg/mL) was added, the mixture was incubated for 1 h at 55 °C. After hydrolysis, 60–70 mg of solid buffer carbonate/bicarbonate was added to alkalise the sample, and the liquid/liquid extraction was carried out twice with 3 mL of TBME. After centrifugation, the organic layer was combined and transferred. After evaporation, the residue was either derivatized with TMIS reagent (MSTFA/NH4I/ethanethiol, 1000:2:3, v:w:v) by heating for 20 min at 75 °C and injected into the GC–MS or reconstituted in methanol for LC–MS analysis.

### GC-QTOF-MS

The GC-QTOF-MS analyses for potential MD metabolites in incubation experiments were performed on an GC-QTOF 7890B/7250 (Agilent Technologies, Milano, Italy), equipped with an Agilent HP1 column (17 m, 0.20 mm; 0.11 μm) with helium as carrier gas. Injection was performed in split mode (1:10) at 280 °C applying an injection volume of 2 µL. The oven temperature was programmed as 0 min 150 °C, ramped with + 50 °C/min to 240 °C (0 min), + 3 °C/min to 266 °C (0 min), further + 50 °C/min to 320 °C (3 min hold). Electron ionization (EI) was performed at 70 eV with data acquisition in full scan mode (*m/z* 50 to 750).

### LC–MS

Untargeted high resolution accurate mass analysis for the investigation of potential MD metabolites were performed on an Agilent 6550i QToF (Agilent Technologies, Santa Clara, USA) coupled to an 1290 Infinity II HPLC system (Agilent Technologies, Santa Clara, USA). The system was equipped with an Agilent ZORBAX Eclipse Plus Phenyl-Hexyl column (3.0 × 100 mm; 1.8 µm particle size). Column temperature was controlled and held at 30 °C. Water (eluent A, H_2_O:FoOH, 99.9:0.1, *v/v*) and acetonitrile (eluent B, ACN: FoOH 99.9:0.1, *v/v*) were used as eluents. The gradient program started with 2% eluent B increasing linear to 55% in 3 min, then to 95% in 3.5 min, 1.5 min hold and 0.2 min back to 2%. The gradient flow rate of 0.550 mL/min resulted in a run time of 8.20 min and 1.30 min for the column equilibration. Aliquots of 1.0 µL were injected into the system.

The mass spectrometer was operated in auto MS/MS mode using positive ionization (ESI +). Mass range for MS spectra was between *m/z* 100 and *m/z* 1000 (2 spectra/sec), MS^2^ spectra were collected between *m/z* 50 and *m/z* 1000 (3 spectra/sec). Permanently performed mass axis calibration and a high resolution (> 10,000) achieved high mass accuracy. Drying gas flow was set to 11 mL/min at 200 °C, sheath gas flow to 11 mL/min at 375 °C, nebulizer pressure to 35 psi (N_2_), capillary voltage to 3500 V and the nozzle voltage to 500 V.

## Results

### Fish embryo toxicity

**Fig. 1:**
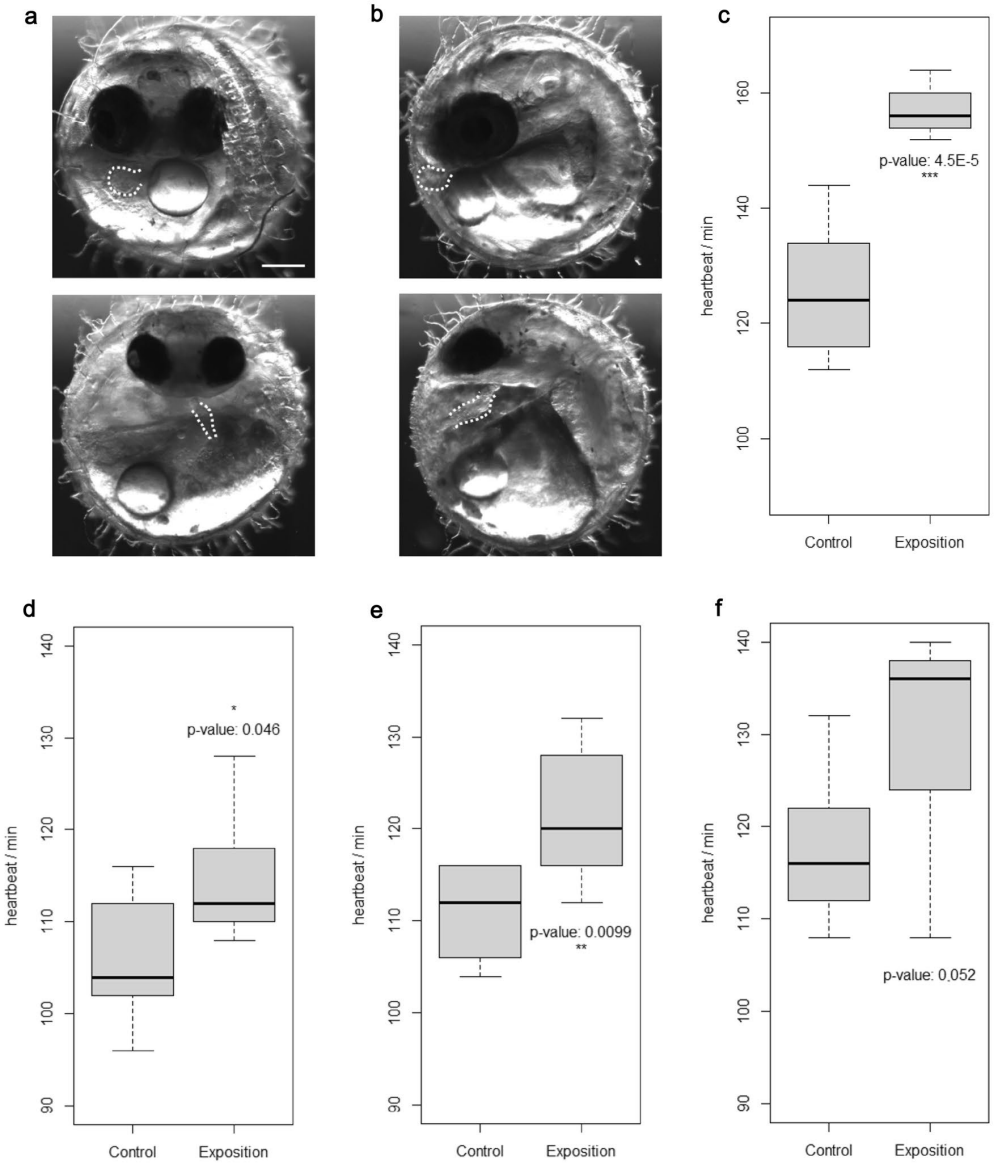
Developmental effects (**a**, **b**) and changes in heart beat rate (**c**–**f**) after exposition to metandienone. **a** Regular heart development of medaka embryo after 8 days of development in frontal view (top); after exposition to 50 µM MD, the heart has a tube-like morphology and contractile structures are nearly absent (bottom). **b** Lateral views as in **a**. Dotted lines either mark the ventricle or the tube-like heart. Scale bar: 200 µM. (**c**) Increased heart frequency after exposition to 10 µM MD for 8 days. **d**–**f** Increased heart frequency after exposition to 10 µM MD for 2 days in three repeated experiments. Significance code of two-tailed Student’s *t* test (unpaired): *p value < 0.05; **p value < 0.01; ***p value < 0.001

Our observations of the embryonic development indicated a developmental malformation of the head, the eyes and the circulatory system in embryos that were exposed for 8 days at the highest concentration tested (50 µM MD, Fig. 1a, b). These individuals showed defects in vessel formation and many blood islands at day 8. We frequently observed curled tails and malformed hearts still beating, but the malformed, tube-like ventricle reduces the heart’s pumping capacity (Supplementary Information, Movies 1–3). Apart from severely affected individuals that frequently died, embryos hatched as expected around day 11.

### Heart beat

An elevated heart beat rate was still detectable in embryos treated with 10 µM MD for 8 days (Fig. 1c; Supplementary Information, movies 1, 2). Since the aim of this study was to evaluate the metabolite production, we incubated the embryos with 10 µM MD for only 48 h (day 6–8) in repeated experiments to reduce the developmental effects. Indeed, the increase in heart beat was no longer of statistical significance (Fig. 1d–f, Table S1). The shortened exposition allowed metabolite analysis and seems appropriate to minimize developmental defects that might interfere with physiology and metabolism. Additional toxicity data should be gained a priori in a long-term exposition when needed.

### Metabolism analysis

#### LC-QTOF-MS/MS analysis

**Fig. 2:**
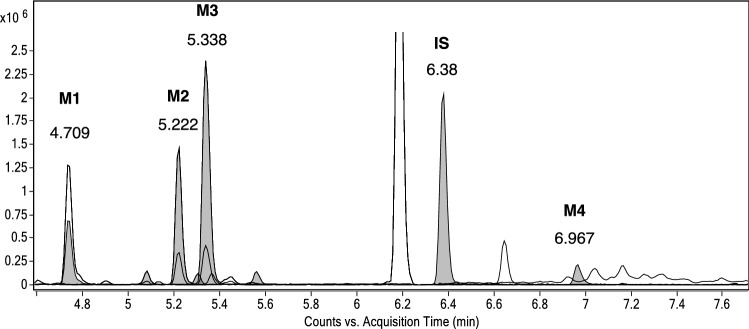
Extracted chromatogram of a sample obtained after 2-day incubation of medaka embryos with 10 µM MD. 6βOH-MD (**M1**, RT 4.709 min), mono-hydroxy MD (**M2**, RT 5.222 min), mono-hydroxy MD (**M3**, RT 5.338 min), testosterone-d3 (internal standard, **IS**, RT 6.38 min), 17β-hydroxy-17α-methyl-5β-androst-1-en-3-one (**M4**, RT 6.967 min)

Sample extract obtained from medaka embryo incubation with MD was diluted and analyzed by LC–ESI–MS(/MS) analysis. Figure 2 shows the typical chromatogram of the samples obtained after incubation of MD with the medaka embryos. Some of the metabolites were identified by comparison with authentic reference material. Detailed information on retention times, molecular ions and mass errors are available as Supplementary Information (Table S2).

**Fig. 3:**
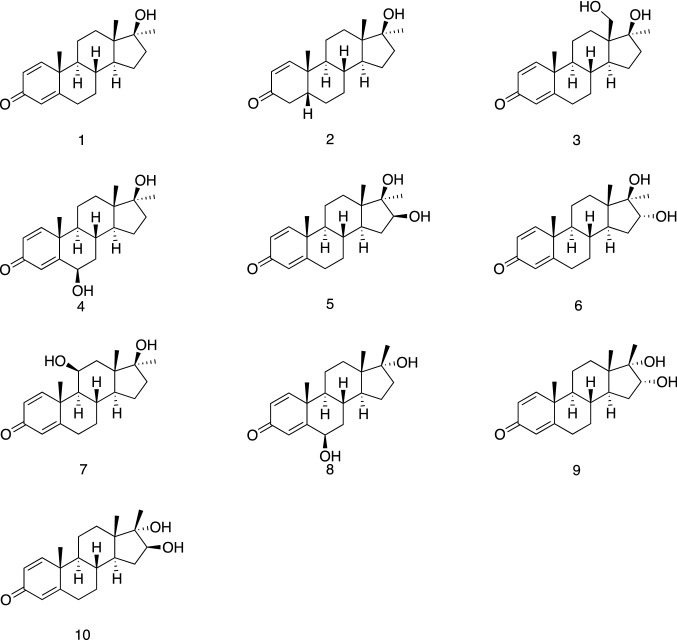
Chemical structure of investigated compounds: MD (1, molecular weight (MW) 300), 17β-hydroxy-17α-methyl-5β-androst-1-en-3-one (2, MW 302), 18-hydroxy metandienone (3, 18OH-MD, MW 316), 6β-hydroxy metandienone (4, 6βOH-MD, MW 316), 16β-hydroxy metandienone (5, 16βOH-MD, MW 316), 16α-hydroxy metandienone (6, 16αOH-MD, MW 316), 11β-hydroxy metandienone (7, 11βOH-MD, MW 316), 6β-hydroxy epimetandienone (8, 6βOH-epiMD, MW 316), 16α-hydroxy epimetandienone (9, 16αOH-epiMD, MW 316), 16β-hydroxy epimetandienone (10, 16βOH-epiMD, MW 316)

Product ion mass spectra were produced and compared with those from the corresponding reference standards. A total of four metabolites were detected by LC–ESI–MS/MS analysis. These include three mono-hydroxylation metabolites (**M1**, **M2**, **and M3**) and one reduced metabolite (**M4**). No epimerized metabolites were found after the incubation. MD has been investigated for years, and its extensive metabolites have been reported via *in vivo* and *in vitro* studies. Figure 3 shows the metabolites of MD after reduction or mono-hydroxylation (Hagedorn et al. 1992; Pozo et al. 2009a; Parr et al. 2012; Schänzer et al. 1991).

**Fig. 4:**
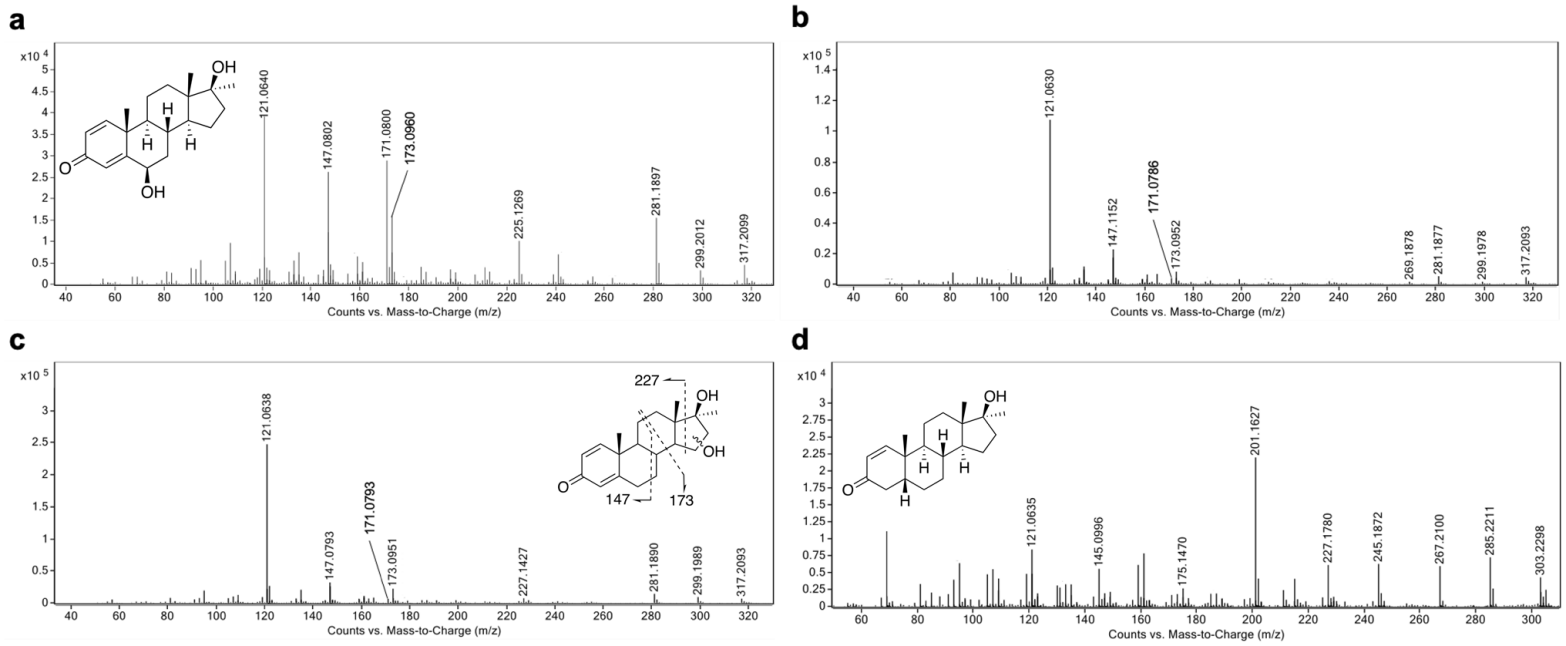
Product ion spectrum of (**a**) 6βOH-MD (RT 4.709 min, **M1**), (**b**) mono-hydroxy MD (RT 5.222 min, **M2**), (**c**) mono-hydroxy MD (RT 5.338 min, **M3**) and (**d**) 17β-hydroxy-17α-methyl-5β-androst-1-en-3-one (RT 6.967 min, **M4**) formed by Medaka embryos incubated with 10 μM MD, LC–ESI–MS/MS, [M + H]^+^  = 317.2028, collision energy 23.1 eV

Structure of **M1** has been assigned to 6βOH-MD by comparison of the data obtained in extracted samples with reference standard (Fig. 2, Fig. 4a). Retention time and product ion mass spectra of **M1** matched well with those from the authentic material of 6βOH-MD (Supplementary Information, Table S3). However, the retention time of **M4** was close to both 17β-hydroxy-17α-methyl-5α-androst-1-en-3-one and 17β-hydroxy-17α-methyl-5β-androst-1-en-3-one. Due to the similarity of the product ion spectra, the stereochemistry at C5 position of **M4** cannot be confirmed based on the LC–ESI–MS/MS analysis.

Metabolites **M1**, **M2** and **M3** have the same molecular mass 317 Da, suggesting a hydroxylation with respect to **MD**. The product ion spectra of **M1**, **M2** and **M3** obtained by LC/ESI–MS(/MS) are shown in Fig. 2a–c. The product ions at *m/z* 299 and 281 were found in all the product ion spectra of these three metabolites, representing consecutive water losses from the molecular ion with *m/z* 317 (Supplementary Information, Tables S3 and S5). The ions at *m/z* 121, 147, 171 and 173 in the product ion mass spectra were commonly observed and discussed for hydroxylated MD metabolites in literature (Pozo et al. 2009a; Musharraf et al. 2013; Kwok et al. 2015), which were also observed in the product ion spectra of **M1**, **M2** and **M3**. The 1,4-dien-3-one structure was identified by the product ions *m/z* 121 and *m/z* 147 (Thevis and Schänzer 2005; Schänzer et al. 2006). The fragment at *m/z* 121 is proposed to result from fissions of C–C bonds between C6–C7 and C9–C10 (Schänzer et al. 2006). However, two different product ions at nominal *m/z* 147 were observed in **M2** and **M3**. As proposed by Thevis et al. and Pozo et al., 147.0793 obtained from **M3** are suggested to result from the A-, B-, and C-rings of 1,4-dien-3-one steroids by fissions of the linkages between C-6 and C-7, C-8 and C-9, and C-11 and C-12, while the fragment at *m/z* 147.1152 from **M2** is generated from C- and D-ring (Thevis and Schänzer 2005; Pozo et al. 2008). This indicates that the intact 1,4-dien-3-one structure has been substantiated in the structure of metabolites **M3**, while the structure of **M2** is proposed to contain the intact D-ring structure from **MD**.

The product ion at *m/z* 225 of **M1** is proposed to generate owing to the loss of 56 Da from the D-ring and two water losses from the quasi molecular ion [M + H]^+^, containing A-, B-, and C-ring (Musharraf et al. 2013). However, this fragment shifts to *m/z* 227 in the product ion spectrum of **M3**, which was also observed for **MD**. Therefore, the location of the hydroxy group in the structure of **M3** is assumed to be at the D-ring.

In the product ion spectrum of **M2** the product ion *m/z* 269 is proposed to be generated due to the neutral loss of formaldehyde (30 Da) from [M + H-H_2_O]^+^, indicating the presence of a hydroxylated methyl residue in the structure of **M2** (Schänzer et al. 2006). Given that **M2** keeps the conserved structure of D-ring in respect to MD, the position of the hydroxylated methyl residue could be assumed at C18 or C19. However, a comparison of spectra and retention time with authentic reference material is necessary for the ultimate confirmation (level 1 confidence according to (Schymanski et al. 2014)).

#### GC-QTOF-MS analysis

**Fig. 5:**
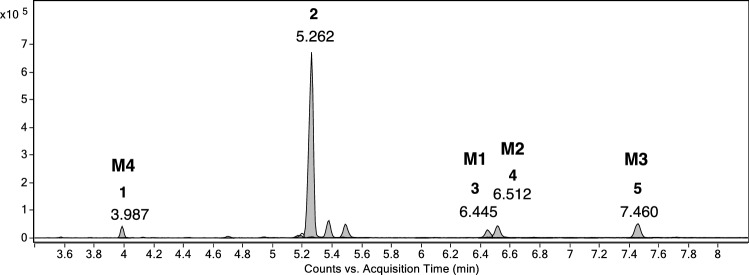
GC-QTOF-MS chromatogram (EICs) after TMIS derivatization showing six main products after 2-day incubation of medaka embryos with 10 µM MD. 17β-hydroxy-17α-methyl-5β-androst-1-en-3-one (1, **M4**, RT 3.987 min), MD (2, RT 5.262 min), 6βOH-MD (3, **M1**, RT 6.445 min), mono-hydroxy MD (4, **M2**, RT 6.512 min), mono-hydroxy MD (5, **M3**, RT 7.460 min)

To have more fragmentation information of the mono-hydroxylated metabolites, and to further confirm the stereochemistry of the C5 structure of **M4**, we performed GC-QTOF-MS in addition. Figure 5 shows the GC-QTOF-MS chromatogram (EICs) after TMIS derivatization of the products. Detailed information on retention times, molecular ions and mass errors are shown in the Supplementary Information (Table S6). The molecular ion of analyte 1 is *m/z* 446. After the comparison of the retention time and mass spectra from analyte 1 with reference materials, analyte 1 was identified as 17β-hydroxy-17α-methyl-5β-androst-1-en-3-one (**M4**, RT 3.987 min). This is also in accordance with the results published before (Schänzer 1996; Loke et al. 2021), which report that almost all of the reduced metabolites produced from **MD** show the 5β-stereochemistry structure.

**Fig. 6:**
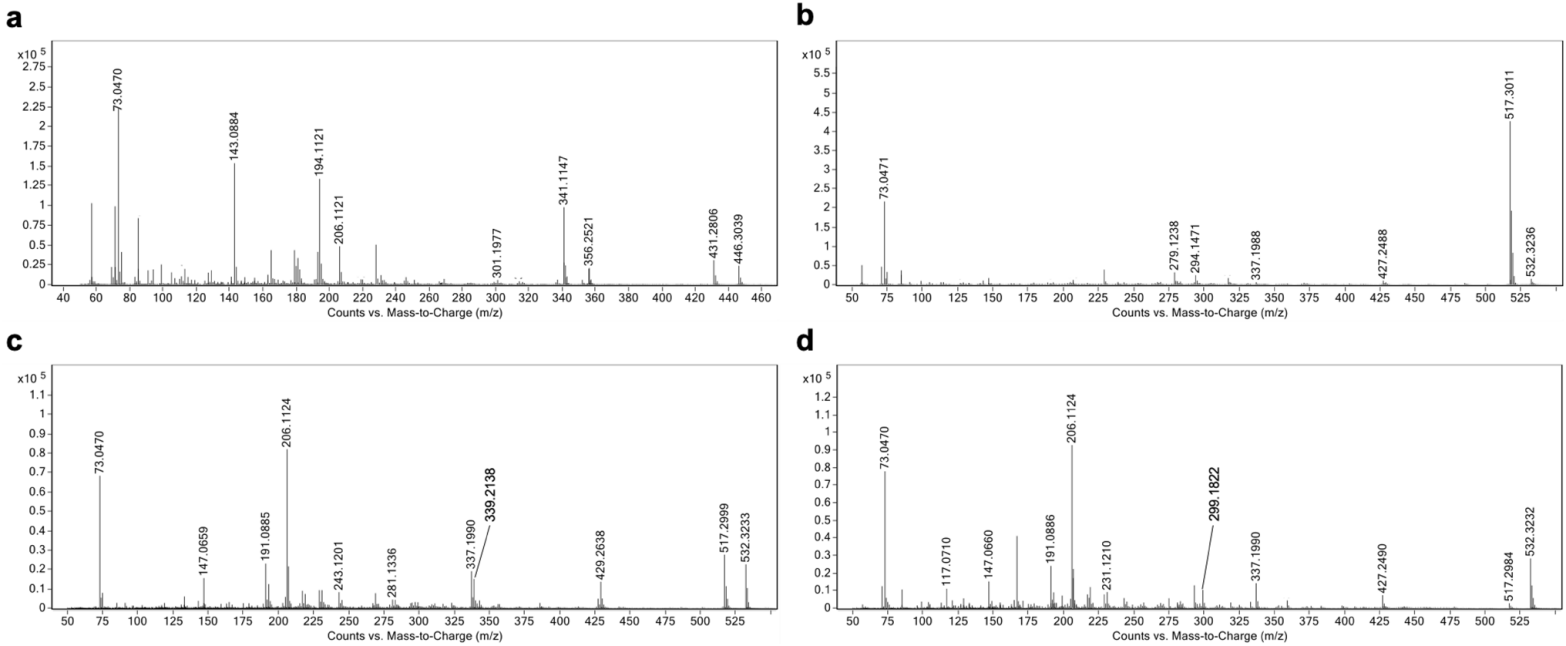
GC-EI-MS spectra of (**a**) 17β-hydroxy-17α-methyl-5β-androst-1-en-3-one (1, **M4**, RT 3.987 min), (**b**) 6βOH-MD (3, **M1**, RT 6.445 min), (**c**) mono-hydroxy MD (4, **M2**, RT 6.512 min), and (**d**) mono-hydroxy MD (5, **M3**, RT 7.460 min), formed by medaka embryos incubated with 10 μM MD

GC-QTOF-MS analysis also showed three mono-hydroxylated metabolites formed by medaka embryos. The molecular ion of tris-TMS-OH-MD derivatives were identified (*m/z* 532). The EI mass spectra of analytes 3, 4 and 5 are depicted in Fig. 6b–d. Analyte 3 (RT 6.445 min) was assigned to **M1** by comparison with authentic reference material (detailed information on fragmentation shown in Supplementary Information, Table S7). Analyte 4 (RT 6.512 min) yielded ions at *m/z* 517 (M^+^ − 15), and *m/z* 337 (M^+^ − 15–2 × 90), which are generated by the losses of ^•^CH_3_ and/or TMS-OH from the molecule (Supplementary Information, Table S8). Only analyte 4 showed the fragment *m/z* 429. This fragment is correlated to the characteristic loss of TMS-O-CH_2_^•^ (103 Da) from the molecular ion (*m/z* 532) discussed for steroids bearing a hydroxymethyl group (Geldof et al. 2014). This is followed by a loss of TMS-OH (90 Da) yielding *m/z* 339 (Parr et al. 2012). As previously proposed, the fragment *m/z* 206 is suggested to arise from the cleavage of the C9-C10 and C7-C8 bonds, comprises the A-ring and the C6 and C7 carbons (Bi and Massé 1992), which indicates the 1,4-dien-3-one structure after enolization by means of trimethylsilylation (Thevis and Schänzer 2007). This fragment was found for analyte 4 and 5. This substantiates an intact structure of A- and B-ring derived from **MD** in both analytes. In comparison to the mass spectrum of 18OH-MD previously reported by Parr, et al., the mass spectrum of analyte 4 is in line with that of 18OH-MD (Fig. 6c) (Parr et al. 2012), providing level 2 confidence in identification. Hence, analyte 4 was postulated to be 18OH-MD (**M2**). However, for full structure confirmation, the analyte 4 still needs further comparison with reference material.

The mass spectrum of analyte 5 (RT 7.460 min) shows ions at *m/z* 427 (M^+^ − 15–90), and *m/z* 337 (M^+^ − 15–2 × 90), indicating two consecutive losses of TMS-OH and ^•^CH_3_ from the molecule (Fig. 6d). The ion at *m/z* 299 is proposed to result from the loss of D-ring (Pozo et al. 2009b). Therefore, the combination of fragment ions *m/z* 206 and *m/z* 299 showed a conserved structure of A-, B-, and C-ring derived from **MD**. The fragment ion *m/z* 231 is proposed to originate from the cleavage of D-ring, which is incremented by 88 Da from the typical fragment of 17-methyl steroids *m/z* 143 due to the additional hydroxy group in the D-ring after TMS-derivatization (Parr et al. 2012). This supports the assumption that the hydroxy group in analyte 5 is located at the D-ring. Accordingly, the mass spectrum of analyte 5 closely resembles that of 16OH-MD (Fig. 6d).

In combination with the data from LC–ESI–MS/MS, analyte 4 was allocated to **M2**, tentatively assigned as 18OH-MD, while analyte 5 was proposed to correspond to **M3**, which was postulated to be 16OH-MD. However, for ultimate confirmation of the structures, but also stereochemical assignment of 16-hydroxylation, comparison with authentic reference materials is necessary.

## Discussion

Fish embryo toxicity protocols were designed for a simple evaluation of few endpoints like egg coagulation, tail morphology or heart beat rate, but further endpoints can and should be integrated in these protocols to detect more specific effects (exemplified for *Danio rerio* by von Hellfeld et al. 2021). In line with this, we aimed to add the metabolic analysis to established toxicology protocols to evaluate metandienone and the production of its metabolites in an assay using medaka embryos. In initial experiments with 1–50 µM MD and exposition from early blastula stage onwards, we detected acute developmental toxicity in medaka embryos (Fig. 1a, b) and the possibility to extract metabolites from the experimental setting. With progressing embryonic development, the number of metabolites was increasing. This was expected due to the formation of tissues and organs and a progressing complexity in cellular pathways. The liver as the main metabolizing organ is formed between day 2 and 4 of embryonic development (Iwamatsu 2004), but it is not known when the full metabolic capacity is established. Only recently, the adult metabolome of the medaka has been analyzed with respect to genomic variation in inbred strains (Soergel et al. 2021). From our experiments with MD as only test substance, we propose to evaluate the metabolic capacity in embryos close before hatching that is day 8 of development at 26 °C.

The d-rR.YHNI strain has a high fecundity but no phenotypic markers for chromosomal sex (Scholz et al. 2003), so embryos of mixed sexes were tested. The choice of 21 embryos per 10-mL medium (approx. 500 µL per embryo) showed the possibility to faithfully extract metabolites from the supernatant. We observed no differences in the metabolite spectrum collecting the supernatants on day 8 or day 10 after 2-day exposition/ metabolization time respectively. Therefore, an incubation from 6 to 8 days of development was established as standard protocol. At 8 dpf, no embryos hatched and the collection of the medium needs no further precaution than pipetting without aspirating the eggs.

Day 8 of medaka development seems also a valid time point to evaluate effects on development and heart beat in medaka. The heart develops from a linear tube at day 2, then loops at the atrioventricular boundary at day 6 and finally shows the pericardial cavity surrounding the heart at day 7–8 (Iwamatsu 2004). Recently, a detailed analysis of medaka (and zebrafish) heart rate proposed 102 h post-fertilization (which equals approx. 4.25 dpf) as possible endpoint in an automated screen (Gierten et al. 2020). Nevertheless, data were evaluated until 7 dpf at 28 °C by Gierten et al., a developmental stage comparable to the present study using 8 dpf medaka embryos at 26 °C. In the future, a consensus on experimental conditions regarding temperature and developmental stage would facilitate a comparison between studies. We would favour an early time point analyzing development and toxicity as well as an advanced one in the view that metabolic activity develops relatively late.

In the individuals exposed to 10 µM MD, we stated the tendency of increased heart beat after 2 days of exposition, but with merely significant values. The increase was in the range of an expected increase potentially caused by an elevated temperature of 1 °C. Since the experimental setting excludes such temperature changes, an elevated metabolism and oxygen consumption might have been a consequence of an anabolic effect leading to the elevated heart beat rate. Alternatively, sub-microscopic effects might have changed the anatomy of the developing heart during the 2-day exposition. In adult strength athletes, a 6-week AAS cycle reversibly increased the left ventricle mass and reduced the ejection volume by 5% (Smit et al. 2021). In view of the fact that the analysis of detailed cardiotoxic effects was beyond the scope of this study, we cannot unambiguously define the elevated heart beat rate in 10 µM MD treated individuals as anabolic effect. To summarize our observations regarding this embryonic test system, we conclude that the absence of visible toxicological effects seems appropriate to investigate medaka embryos as a possible alternative to common human *in vivo* studies and discuss the identified MD metabolites below.

Medaka embryos were exposed to metandienone at developmental stages when liver is already formed. We analyzed the incubation medium for metabolites by LC–ESI–MS/MS and GC-QTOF-MS. The results obtained show that the embryos produced four metandienone metabolites. Those were postulated to be 6β-hydroxy-metandienone (**M1**), tentatively 18OH-MD (**M2**), tentatively 16OH-MD (**M3**) and 17β-hydroxy-17α-methyl-5β-androst-1-en-3-one (**M4**). Since its first detection in human urine samples in 1980, 6βOH-MD is one of the metandienone target metabolites investigated in routine doping analysis (Dürbeck and Büker 1980). The other three metabolites generated by the medaka embryos are also known to be excreted in humans. 17β-hydroxy-17α-methyl-5β-androst-1-en-3-one was first found after MD administration in human urine in 1991 (Schänzer et al. 1991). While 16OH-MD was first detected as a phase I metabolite in horse urine in 1992, administration studies identified 16OH-MD and 18OH-MD as human urinary metandienone metabolites later (Hagedorn et al. 1992; Parr et al. 2012). Although full confirmation of the two mono-hydroxylated metabolites M2 and M3 was not possible due to missing reference material, the comparison of the data obtained with previous results allows for our postulation (Parr et al. 2012). Thus, level 2 confidence in identification is achieved. The existing *in vitro* models used for biotransformation studies comprise liver microsomes, hepatic S9 fraction, hepatocellular lines, primary hepatocytes and isolated recombinant enzymes or fission yeast strains. With the latter two models, it is possible to examine single steps in metabolic pathways. With regard to metandienone, those models have been applied to study hydroxylation of metandienone itself or its metabolites by different cytochrome P450 enzymes (Rendic et al. 1999; Parr et al. 2012) or glucuronidation by UDP-glucuronosyltransferases (Kuuranne et al. 2003). In contrast, liver microsomes and primary cells or cell lines possess many different metabolic enzymes, therefore allowing for sequential reactions. Mazzarino et al. showed the formation of five metabolites by human liver microsomal incubation with metandienone, amongst them 6β-hydroxy-metandienone (**M1**) as well as 16OH-MD (**M3**) (Mazzarino et al. 2018). Strikingly, the liver microsomal incubation generated 13 metabolites if metandienone was co-incubated with 18-nor-metandienone, but they did not observe neither 18OH-MD (**M2**), nor 17β-hydroxy-17α-methyl-5β-androst-1-en-3-one (**M4**) detected in the present study. Primary bovine hepatocytes formed in total five metandienone metabolites, including **M1** and **M4** (Hooijerink et al. 1998), while a more complex metabolite pattern, including **M4** and the known long-term metabolites was observed in HepG2 cells incubated with metandienone (Zschiesche et al. 2021). A possible explanation for the different outcome might by the prolonged incubation in the latter study (up to 14 days versus 24 h). Comparing the *in vitro* biotransformation studies and the medaka embryo model with the human *in vivo* metandienone metabolism, the results reveal that there are slight differences and that none of the model systems is able to reproduce the *in vivo* drug metabolism in its entirety. In comparison to the *in vitro* models, the medaka embryo model allows for a simultaneous analysis of biotransformation and potential toxicity.

In the present study, we detected no metandienone metabolites resulting from 17-epimerization. Moreover, with regard to doping analysis, two metabolites, namely 17α/β-hydroxymethyl-17β/α-methyl-18-nor-androst-1,4,13-trien-3-one are of importance allowing for a significant prolongation to detect a metandienone abuse (Schänzer et al. 2006). In contrast, we detected none of the two long-term metabolites in the present study. As postulated, a prerequisite for the formation of these metabolites as well as the 17-epimerization is the formation of sulfo-conjugated intermediates (Schänzer et al. 1992; De Brabandere et al. 1997). With the present data, a statement on possibly formed sulfo- or glucurono-conjugated metabolites is not possible as we analyzed free, non-conjugated compounds solely. A previous study investigating the metabolism of benzo[a]pyrene in medaka embryo and larvae showed activity of UDP-glucuronosyltransferases in embryos starting from 1 dpf (Hornung et al. 2007). Only recently, Eide and colleagues compared the expression of biotransformation enzymes including sulfotransferases in five teleost fish models among them *Oryzias latipes* (Eide et al. 2021). Unfortunately, the development-dependent expression of those enzymes could only be investigated for zebrafish and stickleback, not for medaka due to missing transcriptomics data. Hence, further investigations on this should include the analysis of intact phase II metabolites generated by medaka embryos.

Based on the comparable outcomes, medaka embryos could serve as an alternative model to identify potential metabolites for human biotransformation of doping-relevant compounds. In the past few years, a couple of studies investigated the zebrafish (*Danio rerio*) as species to model human metabolism of different doping-related compound classes (e.g., anabolic steroids, stimulants, cannabimimetics, secretagogues) (de Souza Anselmo et al. 2017; Sardela et al. 2018, 2020; Matos et al. 2021). The so-called zebrafish water tank model seems to be a promising model for metabolism studies. As this model uses adult zebrafish, every compound treatment representing an animal experiment and therefore needs, however, approval by responsible governing animal welfare authorities.

In contrast, the medaka embryo model investigated in the present study is a non-animal *in vivo* test system for which no approval is required. Further studies are necessary to substantiate the usability of the medaka embryo as a model for human biotransformation. Still, strong expertise and facilities are a prerequisite to address this topic but if proven, this model would contribute to reduce and replace animal experiments as defined by the 3R principle of animal welfare. Beside this, in contrast to the zebrafish, medaka offers another interesting feature that genotypic sex can be easily identified, which might be of interest for future studies.

## Supplementary Information

Below is the link to the electronic supplementary material.

Supplementary file1 (AVI 4082 kb)

Supplementary file2 (AVI 1902 kb)

Supplementary file3 (AVI 2381 kb)

Supplementary file4 (PDF 289 kb)
