# Supplementary material for: Medaka embryos as a model for metabolism of anabolic steroids
Source: Arch Toxicol. 2022 Mar 30;96(7):1963–74. doi: 10.1007/s00204-022-03284-4 (PMC9151555; doi:10.1007/s00204-022-03284-4)
Supplement: Supplementary file 4 — Supplementary file4 (PDF 289 kb) [file 204_2022_3284_MOESM4_ESM.pdf]

Archives of Toxicology

Medaka embryos as a model for metabolism of anabolic steroids

Lingyu Liu[1], Leonie Hobohm[2], Felix Bredendiek[1,3], Alexander Froschauer[2], Oliver Zierau[2], Maria Kristina Parr[1], Annekathrin M. Keiler[2,4,#]

[1] Institute of Pharmacy, Pharmaceutical and Medicinal Chemistry, Freie Universität Berlin, Königin-Luise-Straße 2+4, 14195 Berlin, Germany
[2] Environmental Monitoring & Endocrinology, Faculty of Biology, Technische Universität Dresden, Zellescher Weg 20b, 01217 Dresden, Germany
[3] Core Facility BiosupraMol, Department of Biology, Chemistry, Pharmacy, Freie Universität Berlin, Berlin, Germany

[4] Institute of Doping Analysis & Sports Biochemistry, Dresdner Str. 12, 01731 Kreischa, Germany

# corresponding author: a.keiler@idas-kreischa.de; ORCID 0000-0002-2157-4711

Heartbeat frequency

Table S1 Statistical significance of the difference between heartbeat frequency of the exposition group and their respective control group (* $P \leq 0.05$).

No.	Exposition duration [d]	MD [µM]	control	Shapiro-Wilk test (*P*-value)	Student's t test (*P*-value)
**#2020-5**	8		x	0.361	0.0004*
**#2020-6**	8	10		0.624	
**#MD21-3**	8	10		0.752	4.5E-5*
**#MD21-4**	8		x	0.482	
**#MD21-5**	2	10		0.292	0.046*
**#MD21-6**	2		x	0.647	
**#MD21-7**	2	10		0.404	0.0099**
**#MD21-8**	2		x	0.064	
**#MD21-15**	2	10		0.117	0.052
**#MD21-16**	2		x	0.195	

Movie 1 Frontal view of medaka embryo 8 days post fertilization. Normal development in 0.1% DMSO. The blood flow from atrium to ventricle is easily observed. The ventricle is located on the embryo's right side (on the left in this view).

Movie 2 Frontal view of medaka embryo 8 days post fertilization exposed to 50 µM metandienone for 2 days. Detrimental effects on heart development are recognized as a tube-like heart, a developmental stage similar to 4 days of normal development.

Movie 3 Lateral view of the same embryo as in Movie 1.

LC-QTOF-MS/MS analysis

Table S2 Retention times (LC-ESI-MS/MS), elemental composition, mass errors (Δ*m/z*) of the metandienone metabolites generated by medaka embryos. * Tentative assignment

ID	Elemental Composition	$[M+H]^+$ (calc)	$[M+H]^+$ (exp)	Δ*m/z* [ppm]	RT [min]	Assignment
**M1**	$C_{20}H_{28}O_3$	317.2111	317.2112	-0.32	4.709	Compound 4
**M2**	$C_{20}H_{28}O_3$	317.2111	317.2100	-3.47	5.222	Compound 3*
**M3**	$C_{20}H_{28}O_3$	317.2111	317.2105	-1.89	5.338	Compound 5*/ 6*
**M4**	$C_{20}H_{30}O_2$	303.2319	303.2318	0.33	6.967	Compound 2

Table S3 Postulated fragments, mass errors (Δ*m/z*) for metabolite 6βOH-metandienone **M1** (LC-ESI-MS/MS).

Fragment	[M+H]+ (calc)	[M+H]+ (exp)	Δ*m/z* [ppm]	Rings involved
$[M+H]^+$	317.2111	317.2112	0.32	A-B-C-D
$[M+H-H_2O]^+$	299.2006	299.2012	2.01	A-B-C-D
$[M+H-2H_2O]^+$	281.1900	281.1897	-1.07	A-B-C-D
$[M+H-3H_2O]^+$	263.1794	263.1793	-0.38	A-B-C-D
$[M+H-H_2O-74Da]^+$	225.1274	225.1269	-2.22	A-B-C
$[C_{12}H_{13}O]^+$	173.0961	173.0960	-0.58	A-B
$[C_{12}H_{11}O]^+$	171.0804	171.0800	-2.34	A-B
$[C_{10}H_{11}O]^+$	147.0804	147.0802	-1.36	A-B
$[C_8H_9O]^+$	121.0648	121.0640	-6.61	A

Table S4 Postulated fragments, mass errors (Δ*m/z*) for metabolite **M2** (tentatively 18OH-metandienone; LC-ESI-MS/MS).

Fragment	[M+H]+ (calc)	[M+H]+ (exp)	Δ*m/z* [ppm]	Rings involved
$[M+H]^+$	317.2111	317.2100	-3.47	A-B-C-D
$[M+H-H_2O]^+$	299.2006	299.1978	-9.36	A-B-C-D
$[M+H-2H_2O]^+$	281.1900	281.1877	-8.18	A-B-C-D
$[M+H-H_2O-CH_2O]^+$	269.1900	269.1878	-8.17	A-B-C-D
$[C_{12}H_{13}O]^+$	173.0961	173.0952	-5.20	A-B
$[C_{12}H_{11}O]^+$	171.0804	171.0786	-10.52	A-B
$[C_{11}H_{15}]^+$	147.1168	147.1152	-10.88	C-D
$[C_8H_9O]^+$	121.0648	121.0630	-14.87	A

Table S5 Postulated fragments, mass errors (Δ*m/z*) for metabolite **M3** (tentatively 16OH-metandienone; LC-ESI-MS/MS).

Fragment	[M+H]+ (calc)	[M+H]+ (exp)	Δ*m/z* [ppm]	Rings involved
$[M+H]^+$	317.2111	317.2105	-1.89	A-B-C-D
$[M+H-H_2O]^+$	299.2006	299.1989	-5.68	A-B-C-D
$[M+H-2H_2O]^+$	281.1900	281.1890	-3.56	A-B-C-D
$[C_{16}H_{19}O]^+$	227.143	227.1427	-1.32	A-B-C
$[C_{12}H_{13}O]^+$	173.0961	173.0951	-5.78	A-B
$[C_{12}H_{11}O]^+$	171.0804	171.0793	-6.43	A-B
$[C_{10}H_{11}O]^+$	147.0804	147.0793	-7.48	A-B
$[C_8H_9O]^+$	121.0648	121.0638	-8.26	A

GC-EI-MS analysis

Table S6 Retention times (GC-QTOF-MS), elemental composition, molecular ions ([M]•+) and mass errors (Δ*m*/z) of the per-TMS derivatives of metandienone metabolites generated by medaka embryos. * Tentative assignment

Analyte	Elemental Composition	$[M]^{\bullet+}$ (calc)	$[M]^{\bullet+}$ (exp)	Δ*m/z* [ppm]	RT [min]	Assignment
1	$[C_{26}H_{46}O_2\ Si_2]^{\bullet+}$	446.3031	446.3039	1.79	3.987	Compound 2 (**M4**)
3	$[C_{29}H_{52}O_3\ Si_3]^{\bullet+}$	532.3219	532.3236	3.19	6.445	Compound 4 (**M1**)
4	$[C_{29}H_{52}O_3\ Si_3]^{\bullet+}$	532.3219	532.3233	2.63	6.512	Compound 3* (**M2**)
5	$[C_{29}H_{52}O_3\ Si_3]^{\bullet+}$	532.3219	532.3232	2.44	7.460	Compound 5*/ 6* (**M3**)

Table S7 Postulated fragments, mass errors (Δ*m/z*) for metabolite 6βOH-metandienone **M1** (GC-QTOF-MS).

Fragment	[M+H]+ (calc)	[M+H]+ (exp)	Δ*m/z* [ppm]
$[M]^{\bullet+}$	532.3219	532.3236	3.19
$[M-CH_3]^+$	517.2984	517.3011	5.22
$[M-CH_3-TMSOH]^+$	427.2483	427.2488	1.17
$[M-CH_3-2xTMSOH]^+$	337.1982	337.1988	1.78
$[TMS]^+$	73.0468	73.0471	4.11

Table S8 Postulated fragments, mass errors (Δ*m/z*) for metabolite **M2** (tentatively 18OH-metandienone; GC-QTOF-MS).

Fragment	[M+H]+ (calc)	[M+H]+ (exp)	Δ*m/z* [ppm]
$[M]^{\bullet+}$	532.3219	532.3233	2.63
$[M-CH_3]^+$	517.2984	517.2999	2.90
$[M-CH_2-TMSO]^+$	429.2645	429.2638	-1.63
$[M-CH_3-2xTMSOH]^+$	337.1982	337.1990	2.37
$[M-CH_2-TMSO-TMSOH]^+$	339.2139	339.2138	-0.29
$[TMS]^+$	73.0468	73.0470	2.74

Table S9 Postulated fragments, mass errors ($\Delta m/z$) for metabolite **M3** (tentatively 16OH-metandienone, GC-QTOF-MS).

**Fragment**	**[M+H]+ (calc)**	**[M+H]+ (exp)**	**$\Delta m/z$ [ppm]**
$[M]^{\bullet+}$	532.3219	532.3232	2.44
$[M-CH_3]^+$	517.2984	517.2984	0
$[M-CH_3-TMSOH]^+$	427.2483	427.2490	1.64
$[M-CH_3-2xTMSOH]^+$	337.1982	337.1990	2.37
$[TMS]^+$	73.0468	73.0470	2.74
